# Supplementary figures and images for: Purine nucleosides replace cAMP in allosteric regulation of PKA in trypanosomatid pathogens
Source: eLife. 2024 Mar 22;12:RP91040. doi: 10.7554/eLife.91040 (PMC10959531; doi:10.7554/eLife.91040)

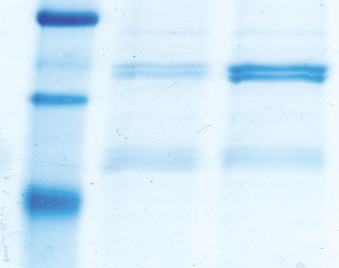

Supplement: Figure 1—figure supplement 1—source data 1. [file elife-91040-fig1-figsupp1-data1.zip › Figure 1-figure supplement 1-source data 1/Figure 1-figure supplement 1-source data 1_Tb.tiff]

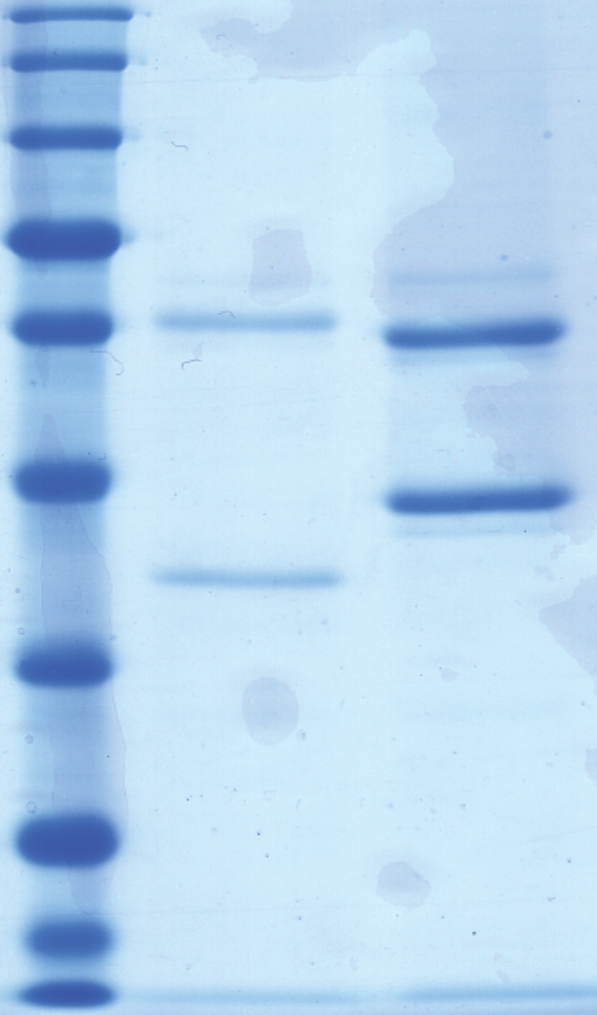

Supplement: Figure 1—figure supplement 1—source data 1. [file elife-91040-fig1-figsupp1-data1.zip › Figure 1-figure supplement 1-source data 1/Figure 1-figure supplement 1 - source data 1_LdTc.tiff]

Figure 1-figure supplement 1-source data 2: Original coomassie stained gels - labelled

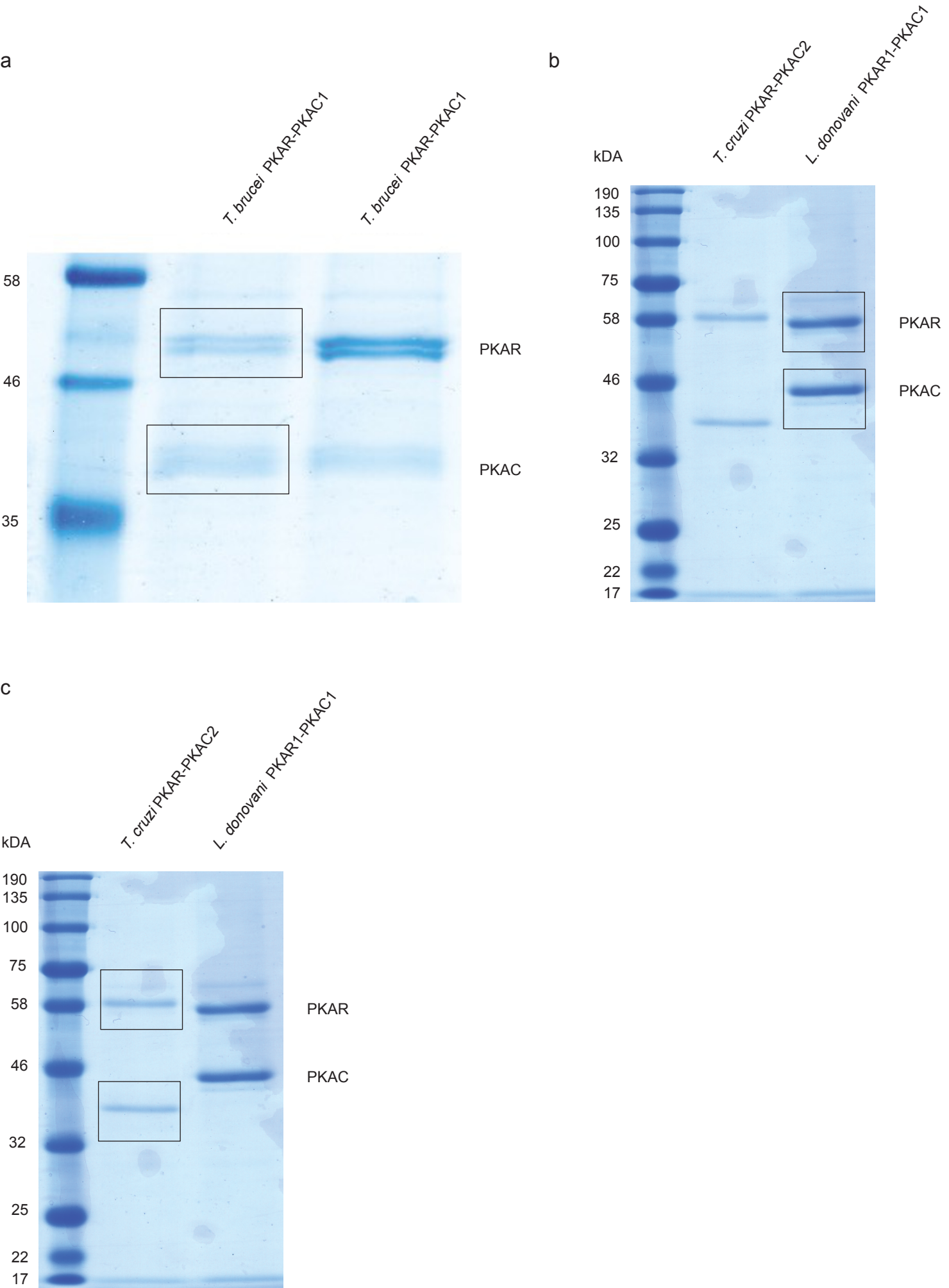

Supplement: Figure 1—figure supplement 1—source data 2. [file elife-91040-fig1-figsupp1-data2.pdf]

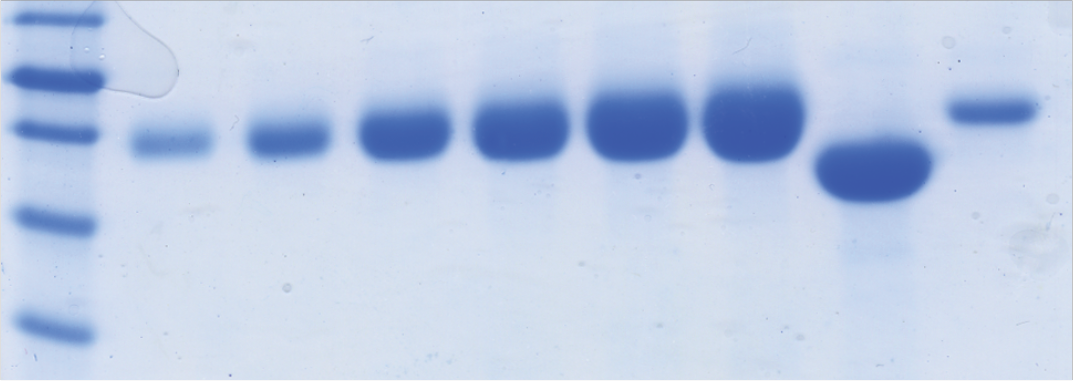

Supplement: Figure 1—figure supplement 2—source data 1. [file elife-91040-fig1-figsupp2-data1.zip › Figure 1-figure supplement 2-source data 1/Figure 1-figure supplement 2-source data 1.tiff]

Figure 1-figure supplement 2-source data 2: Original coomassie stained gel - labelled

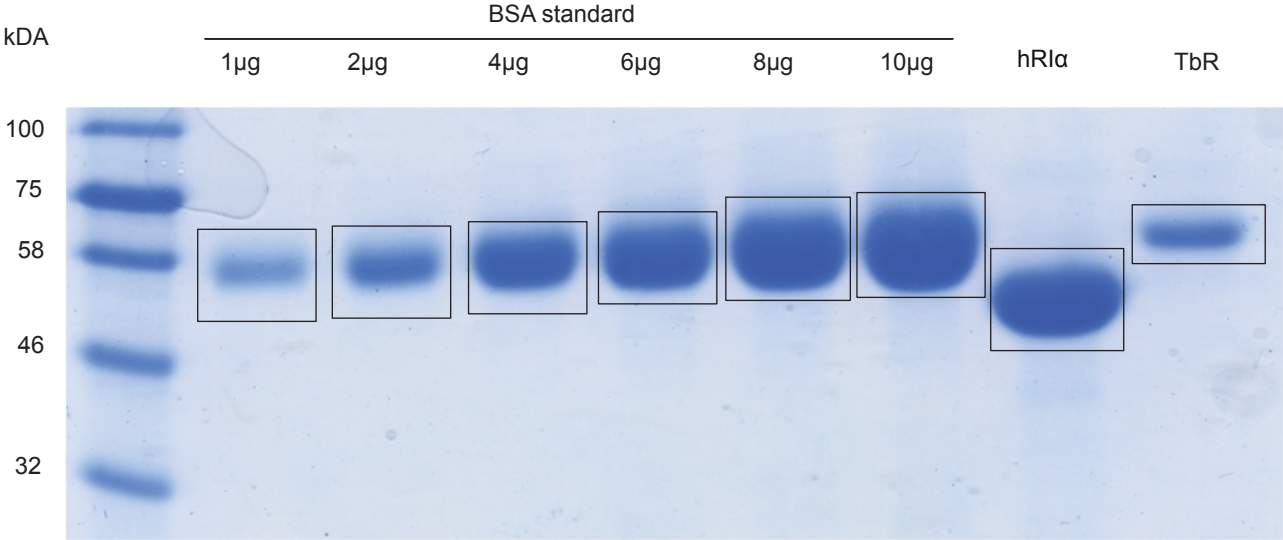

Supplement: Figure 1—figure supplement 2—source data 2. [file elife-91040-fig1-figsupp2-data2.pdf]

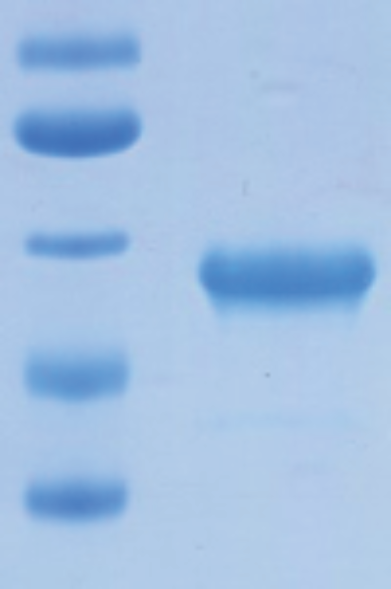

Supplement: Figure 2—figure supplement 1—source data 1. [file elife-91040-fig2-figsupp1-data1.zip › Figure 2-figure supplement 1-source data 1/Figure 2-figure supplement 1-source data 1_Tb.tiff]

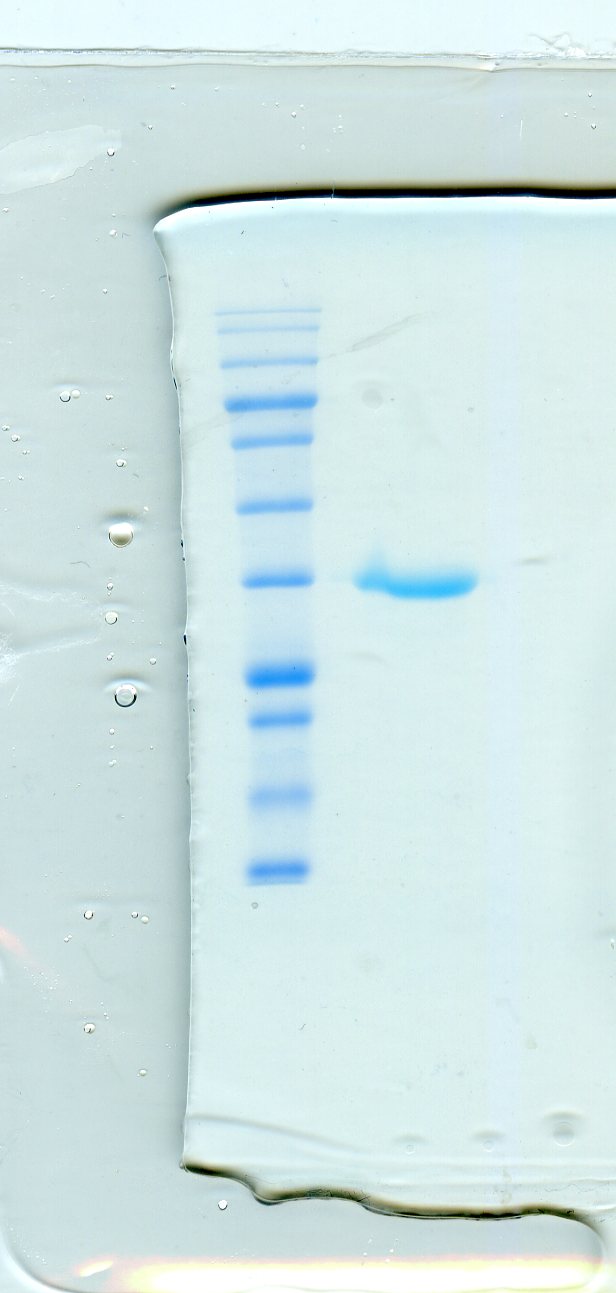

Supplement: Figure 2—figure supplement 1—source data 1. [file elife-91040-fig2-figsupp1-data1.zip › Figure 2-figure supplement 1-source data 1/Figure 2-figure supplement 1-source data 1_Ld.jpg]
