# Supplementary material for: Purine nucleosides replace cAMP in allosteric regulation of PKA in trypanosomatid pathogens
Source: eLife. 2024 Mar 22;12:RP91040. doi: 10.7554/eLife.91040 (PMC10959531; doi:10.7554/eLife.91040)

Figure 2-figure supplement 1-source data 2: Original coomassie stained gels of refolded APO TbPKAR(199-499) and LdPKAR1(200-502) - labelled

a

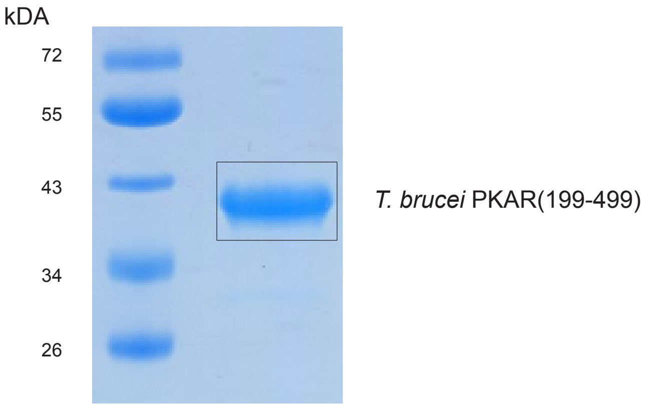

c

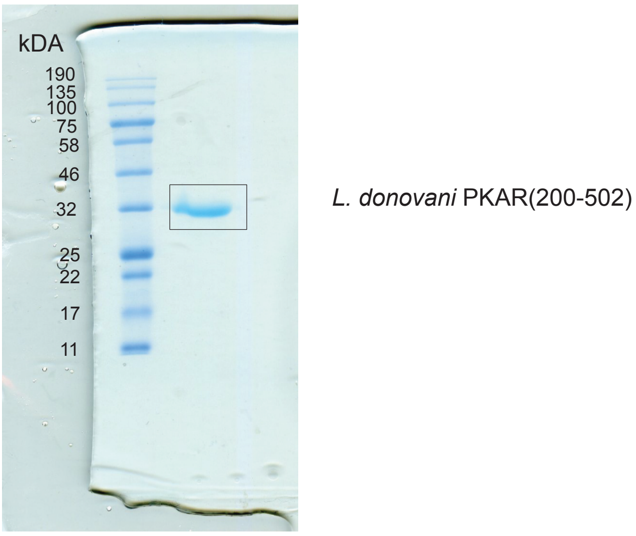

Supplement: Figure 2—figure supplement 1—source data 2. [file elife-91040-fig2-figsupp1-data2.pdf]
