# Supplementary material for: Purine nucleosides replace cAMP in allosteric regulation of PKA in trypanosomatid pathogens
Source: eLife. 2024 Mar 22;12:RP91040. doi: 10.7554/eLife.91040 (PMC10959531; doi:10.7554/eLife.91040)

Figure 6-figure supplement 1-source data 2: Original files for the Western Blot analysis (anti-His, anti PKAC1/2, anti PKAR) - labelled

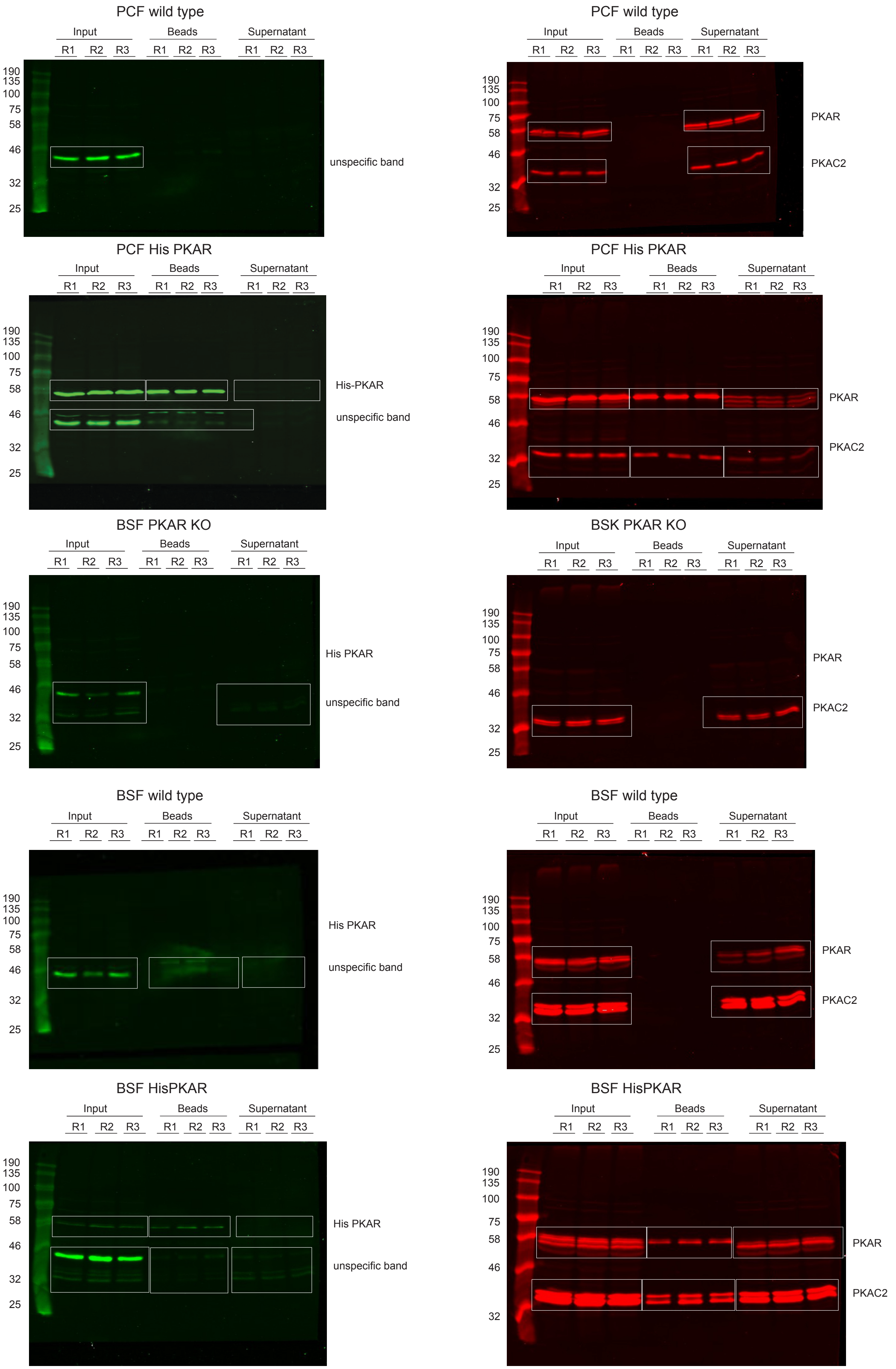

Supplement: Figure 6—figure supplement 1—source data 2. — BSF – blood stream stage, PCF – procyclic stage. [file elife-91040-fig6-figsupp1-data2.pdf]
