# Supplementary material for: Purine nucleosides replace cAMP in allosteric regulation of PKA in trypanosomatid pathogens
Source: eLife. 2024 Mar 22;12:RP91040. doi: 10.7554/eLife.91040 (PMC10959531; doi:10.7554/eLife.91040)
Supplement: Supplementary file 1. [file elife-91040-supp1.docx]

| **PKAR subunit** | **Ligand** | **K_D_ [nM]^1^** | | | **Molar ratio** | | |
| --- | --- | --- | --- | --- | --- | --- | --- |
| human PKARIα | cAMP | 23 | ± | 15 | 1.6 | ± | 0.3 |
|  | Inosine**^3^** | >10^7^ | | |  |  |  |
| TbPKAR(199-499) | Inosine | 18 | ± | 7 | 0.9 | ± | 0.2 |
|  | Guanosine | 150 | ± | 51 | 1.0 | ± | 0.1 |
|  | Adenosine | 825 | ± | 321 | 0.8 | ± | 0.1 |
|  | cAMP^2,3^ | >10^7^ | | |  |  |  |
| LdPKAR1(200-502) | Inosine | 59 | ± | 17 | 1.6 | ± | 0.4 |
|  | Guanosine | 173 | ± | 58 | 1.5 | ± | 0.3 |
|  | Adenosine | 1157 | ± | 226 | 2.0 | ± | 0.1 |
|  | cAMP**^3^** | >10^7^ | | |  |  |  |
| TbPKAR(199-499) E311A, T318R, V319A  (mutant 6) | Inosine | 18 | ± | 10 | 0.5 | ± | 0.1 |
|  | Guanosine | 6 | ± | 3 | 0.7 | ± | 0.1 |
|  | Adenosine | 1100 | ± | 200 | 0.6 | ± | 0.1 |
| TbPKAR(199-499) E435A, N442R, V443A  (mutant 7) | Inosine | 9 | ± | 4 | 0.6 | ± | 0.1 |
|  | Guanosine | 221 | ± | 27 | 0.7 | ± | 0.1 |
|  | Adenosine | 82 | ± | 11 | 0.6 | ± | 0.1 |
| TbPKAR(199-499)  E311A, T318R, V319A  **Y484A, Y485A**  (mutant 8) | Inosine | 1476 | ± | 125 | 0.6 | ± | 0.1 |

**^1^** mean ± SD of ≥ three independent measurements

**^2^** taken from Bachmaier et al. 2019

**^3^** technical limit of MicroCal PeaQ ITC - interpreted as no binding
