# Supplementary material for: Purine nucleosides replace cAMP in allosteric regulation of PKA in trypanosomatid pathogens
Source: eLife. 2024 Mar 22;12:RP91040. doi: 10.7554/eLife.91040 (PMC10959531; doi:10.7554/eLife.91040)
Supplement: Supplementary file 2. [file elife-91040-supp2.docx]

|  | ***T.brucei* PKAR** (inosine) | ***T.brucei* PKAR:**  **E311A,T318R,V319A**  (cAMP – siteA  inosine - siteB) | ***T.cruzi* PKAR** (inosine) |
| --- | --- | --- | --- |
| PDB | 6FLO | 6H4G | 6HYI |
| Cell dimensions |  |  |  |
| a, b, c (Å) | 67.46, 71.55,122.82 | 64.67, 71.20, 121.40 | 40.74, 88.53,46.02 |
| α, β, γ (°) | 90, 90, 90 | 90, 90, 90 | 90, 98.28, 90 |
| Data collection |  |  |  |
| Resolution Range (Å) | 49.08 - 2.14  (2.22 - 2.14) | 30.21 - 2.14  (2.22 - 2.14) | 44.26 - 1.40  (1.45 - 1.40) |
| Space group | P 21 21 21 | P 21 21 21 | P 1 21 1 |
| X-ray wavelength (Å) | 2.075 | 1.34 | 0.9792 |
| Unique reflections | 30009 (1997) | 31336 (3027) | 63354 (6238) |
| Reflections used for R-free | 1998 (119) | 2000 (194) | 3536 (340) |
| Multiplicity | 50 | 3.7 | 6.5 |
| Completeness (%) | 97.4 | 98.9 | 99.3 |
| Mean I/σ (I) | 25.7 (1.0) | 10.4 (1.2) | 11.7 (1.0) |
| Copies per ASU | 2 | 2 | 1 |
| R_merge_ | 0.15 (2.03) | 0.20 (1.78) | 0.069 (1.70) |
| CC1/2 | 99.5 (49.1) | 99.5 (47.2) | 99.7 (48.2) |
| Refinement statistics |  |  |  |
| R_work_ (%) | 0.2354 (0.5243) | 0.2304 (0.3318) | 0.1803 (0.3538) |
| R_free_ (%) | 0.2676 (0.4982) | 0.2987 (0.3907) | 0.2011 (0.3614) |
| Average B factor (A^2^) |  |  |  |
| Protein atoms | 34.04 | 21.66 | 24.26 |
| Solvent atoms | 37.72 | 25.34 | 38.09 |
| Ligand atoms | 33.36 | 18.06 | 18.57 |
| Ramachandran plot (%) |  |  |  |
| Favored/Allowed | 98/2 | 99/1 | 98/2 |
| Outliers | 0 | 0 | 0 |

*Values in parentheses are for highest-resolution shell.
