# Supplementary material for: Purine nucleosides replace cAMP in allosteric regulation of PKA in trypanosomatid pathogens
Source: eLife. 2024 Mar 22;12:RP91040. doi: 10.7554/eLife.91040 (PMC10959531; doi:10.7554/eLife.91040)
Supplement: Supplementary file 4. [file elife-91040-supp4.docx]

Primers used in this study:

**pET-DUET TbPKAR(199-499) (expression of TbPKAR(199-499) in E. coli)**

TbPKAR199-499_FW gccaGGATCCGgaaaacctgtattttcagGGATCTGGCCG GAAGCGACG
TbPKAR199-499_RV cattatgcggccGCttaCTTCCTCCCCTCTGCCCT

**pET-DUET TbPKAR(199-499)_mutant 8 (expression of TbPKAR(199-499)_mutant 8 in E. coli)**

TbPKAR_M8_RV atatatGCGGCCGCTTACTTCCTCCCCTCTGCCCTTAAAGTA

GTTTTCAGTTTCGACTGGGCGGCCTCATA

**pET-DUET TcPKAR(200-503) (expression of TcPKAR(200-503) in E. coli)**

TcPKAR200-503_FW agccaGGATCCGgaaaacctgtattttcagGGATCTGGCAG AAACCGCCGCC
TcPKAR200-503_RV cattatgcggccGCTTATACATCATCCACGTACGATG

**pET-DUET LdPKAR1(200-502) (expression of TcPKAR(200-503) in E. coli)**

LdPKAR1200-502_FW TAGTGGATCCGGCCGGGCTCGTCGCCAGACAG

LdPKAR200-502_RV attatgcggccGCCTACGCGCTCTCGTG

**Co-expression of TbPKAR-10xHis and Strep-TbPKAC1 in Lexsy (vectors pLEXSY_I-ble3, pLEXSY_I-neo3)**

5’PKAR SaII ATGCATCTCGAGATGTCTGAAAAGGGAACATCG

3’PKAR NotI TATATAATAGTTTAGCGGCCGCTCAGTGGTGGTGATGGTGGTGGT

GGTGGTGATGGCTACCGCCCTTCCTCCCCTCTGCCCTTAA

5’Strep BamHI CGCGGATCCATGGCTTCGGCTTGGAGCCAC

PKAC1 Rev NotI ACTATGCAATAGTTTAGCGGCCGCCTAAAAACCACGGAATGCAAC

5’Ble BamHI GCCGGATCCACCATGGCCAAGTTGACCAGT

3’Ble SpeI CTAGAACTAGTTCAGTCCTGCTCCTCGGCCA

**Co-expression of LdPKAR1-6xHis and Strep-LdPKAC1 in Lexsy (vectors pLEXSY_I-ble3, pLEXSY_I-neo3)**

LdPKAR_FW ccgcctcgagatgggcagcagccatcaccatcatcaccacagccaggatccggaaaacctgtatttt

cagggatctATGTCCGCGGAAGACACCCCC

LdPKAR_RV gagggcggccgccttaCTGGACCGCCGCCGGGGCACC

LdPKAC1_FW gCCACCAgatctgCCATGGCTTCGGCTtggagccacccgcagttcgaaaaaGCTTCG

TCTGCTGCCAAGGACAGCTGTCCGGCGGA

LdPKAC1_RV AGGAGGGCGGCCGCCTAAAAGCCATTGAATTCCGCCTGC

**Co-expression of TcPKAR-6xHis and Strep-TcPKAC2 in Lexsy (vectors pLEXSY_I-ble3, pLEXSY_I-neo3)**

TcPKAR_FW ccgcctcgagatgggcagcagccatcaccatcatcaccacagccaggatccggaaaacctgtatttt

cagggatctATGTCCGCGGAAGACACCCCC

TcPKAR_RV gagggcggccgccttaCTGGACCGCCGCCGGGGCACC

TcPKAC2_FW gCCACCAgatctgCCATGGCTTCGGCTtggagccacccgcagttcgaaaaaGCTAC

GTTGAAGAATGCACCTGAATTTGTAAAGCCGGACGC

TcPKAC2_RV AGGAGGGCGGCCGCTTAGAACCCAATAAACTCCGCCTGT

**Co-expression of TbPKAR-mutant1 and Strep-TbPKAC1 in Lexsy (vectors pLEXSY_I-ble3, pLEXSY_I-neo3)**

Mutant1_RBC-A_FW GGAGAGCTTGcACTTATGTATCAGACACCAcgTGcTGCCACGGTG

Mutant1_RBC-A_RV CACCGTGGCAgCAcgTGGTGTCTGATACATAAGTgCAAGCTCTCC

Mutant1_RBC-B_FW GGTGAGCTGGcATTCCTTAACAATCACGCCcgTGcAGCAGATGTT

Mutant1_RBC-B_RV AACATCTGCTgCAcgGGCGTGATTGTTAAGGAATgCCAGCTCACC

**Co-expression of TbPKAR-mutant2 and Strep-TbPKAC1 in Lexsy (vectors pLEXSY_I-ble3, pLEXSY_I-neo3)**

Mutant2_RBC-A_FW GGAGAGCTCGcACTTATGTATCA

Mutant2_RBC-A_RV TGATACATAAGTgCGAGCTCTCC

Mutant2_RBC-B_FW AGCTGGcATTTTTAAACAAT

Mutant2_RBC-B_RV ATTGTTTAAAAATgCCAGCT

**Co-expression of TbPKAR-mutant3 and Strep-TbPKAC1 in Lexsy (vectors pLEXSY_I-ble3, pLEXSY_I-neo3)**

Mutant3_RBC-A_FW GTAGGAGAGCTTGAACTTATGTATCAGACACCggtTGTTGCCACG

Mutant3_RBC_A_RV CACCGTGGCAACAaccGGTGTCTGATACATAAGTTCAAGCTCTCCTAC

Mutant3_RBC-B_FW GTGGGTGAGCTGGAATTCCTTAACAATCACGCCgtTGTgGCAGAT

Mutant3_RBC-B_RV ATCTGCcACAacGGCGTGATTGTTAAGGAATTCCAGCTCACCCAC

**Co-expression of TbPKAR-mutant4 and Strep-TbPKAC1 in Lexsy (vectors pLEXSY_I-ble3, pLEXSY_I-neo3)**

Mutant4_RBC-A_FW CAGACACCAcgcGTCGCGACGGTG

Mutant4_RBC-A_RV CACCGTCGCGACgcgTGGTGTCTG

Mutant4_RBC-B_FW AATCACGCCcggGTAGCAGACGTCGTG

Mutant4_RBC-B_RV CACGACGTCTGCTACccgGGCGTGATT

**Co-expression of TbPKAR-mutant5 and Strep-TbPKAC1 in Lexsy (vectors pLEXSY_I-ble3, pLEXSY_I-neo3)**

Mutant5_RBC-A-FW GTAGGAGAGCTCGcACTTATGTATCAGACACCAcgcGTCGCGACGGTG

Mutant5_RBC-A-RV CACCGTCGCGACgcgTGGTGTCTGATACATAAGTgCGAGCTCTCCTAC

Mutant5_RBC-B-FW GGTGAGCTGGcATTTTTAAACAATCACGCCcggGTAGCAGACGTCGTG

Mutant5_RBC-B-RV CACGACGTCTGCTACccgGGCGTGATTGTTTAAAAATgCCAGCTCACC

**Co-expression of human PKARIalpha and mouse PKACalpha (in pETDuet-1)**

5’strep (NdeI) Mm Cα ttGGAATTCCATATGGCTTCGGCTtggagccacccgcagttcgaaaaaGCTGGCAAC

GCCGCCGCCGCCAAGAAGGGC

3’(AvrII)MmCα ttcTGCCTAGGCTAAAACTCAGTAAACTCCTTGCC

6H.tev./HsPKARIaf GCCAGGATCCGGAAAACCTGTATTTTCAGGGATCTATGGAGTCTGGCAGTA

6H.tev/HsPKARIaf attatgcggccGCTCAGACAGACAGTGA
